# Managing clustering effects and learning effects in the design and analysis of multicentre randomised trials: a survey to establish current practice

**DOI:** 10.1186/s13063-020-04318-x

**Published:** 2020-05-27

**Authors:** Elizabeth J. Conroy, Jane M. Blazeby, Girvan Burnside, Jonathan A. Cook, Carrol Gamble

**Affiliations:** 1 Department of Health Data Science, University of Liverpool, a member of Liverpool Health Partners, Liverpool, UK; 2 Liverpool Clinical Trials Centre, University of Liverpool, Liverpool, UK; 3 Centre for Surgical Research, Bristol Biomedical Research Centre, Population Health Sciences, University of Bristol, Bristol, UK; 4 Centre for Statistics in Medicine, University of Oxford, Oxford, UK

**Keywords:** Trials, Clinical Trials Unit, Clinical trial, Randomised controlled trial, Complex intervention, Surgical intervention, Trial design, Trial analysis, Survey, Clustering, Learning

## Abstract

**Background:**

Patient outcomes can depend on the treating centre, or health professional, delivering the intervention. A health professional’s skill in delivery improves with experience, meaning that outcomes may be associated with learning. Considering differences in intervention delivery at trial design will ensure that any appropriate adjustments can be made during analysis. This work aimed to establish practice for the allowance of clustering and learning effects in the design and analysis of randomised multicentre trials.

**Methods:**

A survey that drew upon quotes from existing guidelines, references to relevant publications and example trial scenarios was delivered. Registered UK Clinical Research Collaboration Registered Clinical Trials Units were invited to participate.

**Results:**

Forty-four Units participated (*N* = 50). Clustering was managed through design by stratification, more commonly by centre than by treatment provider. Managing learning by design through defining a minimum expertise level for treatment provider was common (89%). One-third reported experience in expertise-based designs. The majority of Units had adjusted for clustering during analysis, although approaches varied. Analysis of learning was rarely performed for the main analysis (*n* = 1), although it was explored by other means. The insight behind the approaches used within and reasons for, or against, alternative approaches were provided.

**Conclusions:**

Widespread awareness of challenges in designing and analysing multicentre trials is identified. Approaches used, and opinions on these, vary both across and within Units, indicating that approaches are dependent on the type of trial. Agreeing principles to guide trial design and analysis across a range of realistic clinical scenarios should be considered.

## Background

Patient outcomes depend crucially on the treatment provider delivering the intervention. Where there is more than one treatment provider, outcomes observed in patients treated by the same treatment provider may be more similar than those in patients treated by other treatment providers, a phenomenon known as clustering. Whilst treatment providers are often thought of as health professionals, such as general practitioners, nurses, surgeons or therapists, the potential for clustering is also present for treating centre within a clinical trial [1, 2]. In addition to clustering, a change in skill in treatment delivery may be observed over time; specifically, there may be a learning element experienced within one or all of the arms of the study observed during the course of the trial, meaning that trial outcomes may also change and be associated with changes in skill [3]. When comparing interventions within a clinical trial, it is imperative that any trial is designed under a common protocol with regard to treatment delivery, and that the trial is conducted in accordance with this protocol. At trial outset, a researcher may consider the homogeneity of any intervention under examination and the degree to which it is appropriate to standardise these procedures [4]. In extreme cases, where the trial results are questioned by the research community related to the study results, the trial team should be prepared to alleviate any doubts of heterogeneity of treatment effects [5].

Difference in treatment delivery is often considered more of a concern in trials investigating a complex intervention, such as surgery. Trials involving a complex intervention are often criticised because of variability between intervention providers (clustering) but also due to variability over time, often as a result of increased experience (learning) [4]. Recognition, and management as appropriate, of clustering and learning is recommended, and it may have increased relevance within the surgical field, dependent upon the interventions being investigated and their routine use [2, 4, 6–9]. Considering these aspects at trial outset will ensure that any necessary adjustment, to the design or analysis of the study, is applied in a manner appropriate for the intervention under investigation and will support clinical decision-making [5].

Whilst the notion of clustering and learning is familiar to many statisticians, the extent to which these considerations are made, and how, is unknown. A survey to establish current practice for the statistical management of clustering and learning effects in the design and analysis of randomised multicentre trials was undertaken within UK Clinical Research Collaboration (UKCRC) Registered Clinical Trials Units (CTUs) [10]. This survey aimed to ascertain the UK-wide experience of running multicentre studies, in particular those investigating a complex or surgical intervention, in addition to establishing awareness of design issues associated with these studies and levels of concerns around these issues.

## Methods

The survey was delivered at the bi-annual Statisticians Operational Group Meeting in April 2018. Attendees were statistical representatives from each of the UKCRC Registered CTUs [10]. Units that did not have a representative present at this meeting, or did not respond, were contacted via email following the event and invited to participate. Registered CTUs were identified from the network website [10] on 4 January 2018 (*n* = 51, of which 50 were registered at the time of survey; see Supplementary Box 1). As the survey involved professionals and discussions of current practice, no formal ethical approvals were deemed necessary.

### Survey

EJC and CG developed the survey, and GB, JMB and JAC reviewed and provided feedback. The survey was subsequently piloted and revised prior to roll out (Supplementary Box 2).

This survey was developed to establish experience in multicentre trials, in particular those investigating a complex intervention. In order to contextualise the survey content, questions drew upon quotes from existing guidelines, references to relevant publications and example scenarios developed by the study team ([4, 9, 11], Table 1). Questions included concepts such as CTUs’ experience in adjusting for clustering (therapist/surgeon or centre) or time-varying effects (learning curves) and, when a Unit had experience, when and how adjustments are applied. This survey also aimed to establish awareness about design issues in surgery and levels of concern around these issues.

**Table 1:** Example trial scenarios

Scenarios	
A	A trial with a large^a^ sample size, recruiting in several centres, each with multiple treatment providers
B	A trial with a small^b^ sample size, recruiting in several centres, each with multiple treatment providers
C	A trial that recruits within several centres, where treatment providers treat patients across recruiting centres; i.e. treatment provider is not unique to a centre
D	A trial recruiting from several centres, each with multiple treatment providers, investigating a surgical intervention
E	A trial recruiting from several centres, each with multiple treatment providers, investigating substantially different surgical interventions, e.g. a trial comparing surgery to an injection

^a^Centres recruiting at least 10 patients per site

^b^Centres recruiting 2 to 3 patients per site

Questions were analysed and reported by Unit. To represent Unit practice and experience as a whole, Units with multiple responders were combined. In the case that multiple responders from a single Unit provided contradictory answers, for example one responder stated they had experience and another stated they did not, it was assumed that the Unit had the experience. However, due to the nature of the network meeting invites (one per registered CTU), multiple responders from a single CTU were minimised.

### Analysis

Quantitative data from closed questions were analysed using descriptive statistics with standard statistical software (Statistical Analysis Software [SAS®] 9.1.4; SAS Institute Inc., Cary, NC, USA); no formal statistical testing was undertaken.

Free text answers were used to contextualise and illuminate quantitative responses.

To ensure anonymity, each Unit was assigned a project identification number.

## Results

### Unit participation and demographics

Forty-seven of the 50 UKCRC Registered CTUs were represented at the network meeting on 28 April 2018. Of those present, 34 representatives from 31 CTUs (62%) participated. Following the meeting, Units without a completed survey were contacted, of which 13 responded (*n* = 13/19). Supplementary Table 1 provides further details. The overall participation rate of registered Units was 88% (*n* = 44/50). One representative from a newly registered Unit reported lack of experience as a reason for non-participation; reasons were not provided from the remaining five Units.

All responders had a statistical background, with the majority of responders holding a senior or lead at their Unit (senior statistician: *n* = 15/44, 34%; statistical lead: *n* = 13/44, 30%). Supplementary Table 2 provides further details*.*

CTUs listed on the UKCRC Resource Finder [10] as conducting cluster or surgical trials had participation rates of 94% (*n* = 16/17) and 92% (*n* = 33/36) respectively (see Supplementary Table 3). CTUs with a methodological research area in complex interventions participated with a rate of 90% (*n* = 35/39).

Three-quarters of CTUs indicated experience in running trials with a complex intervention (*n* = 32/44, 73%) and two-thirds in running trials with a surgical intervention (*n* = 29/44, 66%), with 25 (57%) indicating experience in both. Seven CTUs stated that their Unit did not have experience in running trials with either type of intervention (*n* = 7/44, 16%). One did not respond to this question (Question 1, Supplementary Table 4).

### Managing effects through design

#### Clustering

Twenty-five Units had undertaken multicentre trials that did not stratify by centre (*n* = 25/44, 57%; see Table 2, Question 2, and Table 3). Common reasons for not stratifying by centre were many centres with few participants (*n* = 19/25, 76%) and expected homogeneity of treatment effect (*n* = 11/25, 44%). Additional reasons for not stratifying by centre included allocation concealment in an open trial; logistical reasons; and grouping centres by region. One responder clearly indicated that this decision was influenced by the nature of the intervention, stating:

“ *… drug trials less effect due to centre compared to say complex or surgical interventions.*” [ID23]

**Table 2:** Methods for managing clustering and learning by design

Question			Category	Response statistics		
				*n*	*N*	*n*/*N*%
2	Does your Unit have any multicentre trials that do not stratify randomisation by centre?		Yes	25	44	57%
			No	18	44	41%
	*See Table**4**for further details.*		No response	1	44	2%
3	In each of the following scenarios, how was the randomisation stratified in trials that your Unit has run? Select all that apply. *See Supplementary Table* *5**for further details.*					
	A	Large sample size,^a^ recruiting in several centres, each with multiple treatment providers	Experience in trial type	39	44	89%
			Centre	34	39	87%
			Treatment provider	3	39	8%
			Both	10	39	26%
			Neither	1	39	3%
			No experience in trial type	4	44	9%
			No response	1	44	2%
	B	Small sample size,^b^ recruiting in several centres, each with multiple treatment providers	Experience in trial type	31	44	70%
			Centre	24	31	77%
			Treatment provider	2	31	6%
			Both	2	31	6%
			Neither	7	31	23%
			No experience in trial type	12	44	27%
			No response	1	44	2%
	C	Recruiting in several centres, where treatment providers treat patients across recruiting centres (treatment provider is not unique to a centre)	Experience in trial type	16	44	36%
			Centre	14	16	88%
			Treatment provider	3	16	19%
			Both	1	16	6%
			Neither	0	16	0%
			No experience in trial type	27	44	61%
			No response	1	44	2%
	D	A trial investigating a surgical intervention, recruiting from several centres, each with multiple treatment providers	Experience in trial type	25	44	57%
			Centre	21	25	84%
			Treatment provider	3	25	12%
			Both	5	25	20%
			Neither	3	25	12%
			No experience in trial type	17	44	39%
			No response	2	44	5%
	E	Recruiting from several centres, each with multiple treatment providers, comparing substantially different interventions e.g. surgery to an injection	Experience in trial type	16	44	36%
			Centre	13	16	81%
			Treatment provider	0	16	0%
			Both	2	16	13%
			Neither	2	16	13%
			No experience in trial type	26	44	59%
			No response	2	44	5%
	In scenarios where Unit has experience, approaches to stratification changes across scenario, i.e. within-Unit variation to stratification		Different approaches across scenarios	20	44	46%
			Same approach across all scenarios	19	44	43%
			No response to Question 3	5	44	11%
4	In the trials run by your Unit, have you defined a minimum level of expertise for the health professionals participating in the trial in terms of:		Treating the condition within the patient population	24	44	55%
			Delivering the trial intervention	31	44	70%
			Setting a minimum professional level of treatment providers	22	44	50%
			*Other approach:*			
			Based on paramedic experience (defined by years in service)	1	44	2%
			Based on surgeon experience (at or beyond a certain level)	1	44	2%
			Centre required to conduct a certain number of operations per year	1	44	2%
			Clinical decision for Chief Investigator	1	44	2%
			Deliverer required to pass surgical and radiotherapy quality assurance	1	44	2%
			Depends on phase of trial—early or pragmatic require different levels	1	44	2%
			In our stepwise study, all therapists were experienced but the intervention was brand new	1	44	2%
			Investigators who define research question are experts in the field and have trained staff to deliver intervention	1	44	2%
			No consistent approach across all our studies.	1	44	2%
			No Unit-wide policy—decided trial by trial depending on intervention and setting	1	44	2%
			Surgeon manuals signed off by ‘senior’ surgeon prior to participation	1	44	2%
			Surgical team led by consultant, who submits video measured for quality assurance, prior to participation	1	44	2%
			These have been implicitly taken as a Chief Investigator and Principal Investigator	1	44	2%
			Training provided to health care professionals in order to participate	1	44	2%
			No, or no response	5	44	11%
5	Has your Unit conducted trials with an expertise-based design, in which participating treatment providers provide only the intervention in which they have expertise?		Yes, when applicable^c^	13	44	30%
			No, with justification	1	44	2%
			No	26	44	59%
			No response	4	44	9%

^a^With centres each recruiting at least 10 patients

^b^With centres each recruiting 2 to 3 patients

^c^We only have one grant application for which we’ve proposed an expertise-based design this year but no prior experience of running a trial with such a design before [ID22]

**Table 3:** Reasons for having multicentre studies that do/do not stratify by centre (Question 2)

Unit has multicentre trials that do not stratify randomisation by centre?	Yes (*N* = 25)		No (*N* = 18)	
*Reason(s) provided*				
Expected homogeneity of treatment effect across centres	11	44%	2	11%
No interest in centre effect	4	16%	1	6%
Lots of centres with few participants per centre	19	76%	1	6%
Not convinced of appropriateness of either fixed or random effect models for centres in the trial	1	4%	0	0%
*Other reason provided*				
Aids in blinding if trial open label	1	4%	0	0%
Balance against other important factors. Centre effect less important in drug trials compared to complex or surgical interventions	1	4%	0	0%
Concern that, in an unblinded trial, stratifying by centre would make it easier to predict the treatment allocated to the next patient [12] 16:405).	1	4%	0	0%
For practical reasons	0	0%	1	6%
Intervention takes place out of hospital	1	4%	0	0%
Large sample size with small/moderate number of centres. We expect balance to be achieved with simple randomisation	1	4%	0	0%
Likely to stratify by geographical region if not by centre	1	4%	0	0%
Randomisation system defaults to stratifying by centre but one example where minimised trial did not. Need to consider balance of resources and avoid confounding. There is a lot of academic debate. e.g. Torgerson.	0	0%	1	6%
Sometimes stratify by region	1	4%	0	0%
Stratified by treatment provider within centres and treatment providers unique within centre	1	4%	0	0%
Undertaken in limited/exceptional circumstances only, e.g. feasibility studies	1	4%	0	0%

One responder who did stratify all the Unit’s trials by centre alluded to concerns regarding potential for unequal distribution of costs across centres:

“*This subject gets a lot of academic debate in some academic circles. But: our randomisation defaults to stratifying by centre; need to balance resources—don’t want to give one too many overheads; balancing avoids confounding; other opinions, such as Torgerson, exist.”* [ID8]

Question 3 asked responders to consider five scenarios (Tables 1, 2 and Supplementary Table 5), in particular their approach to stratifying the randomisation in trials of each type run by their Unit. Responses to Scenario A, of which 39 Units had experience, indicated that most Units when running a trial with a large sample size, with multiple treatment providers per centre each recruiting a minimum of 10 participants, would stratify by centre alone (*n* = 34/39, 87%).

Three would stratify by treatment provider alone (*n* = 3/39, 8%). Seventy percent had experience in running trials like Scenario B, which was the same as Scenario A, only with a small sample size (*n* = 31/44, 70%). As with Scenario A, most Units ran such trials by stratifying by centre alone (*n* = 24/31, 77%) and few by treatment provider alone (*n* = 2/31, 6%).

Responders had less experience running Scenario C trials, trials recruiting in several centres where treatment providers treated patients across centres (*n* = 16/44, 36%). Again, most common was stratification by centre only (*n* = 14/16, 88%), with a greater number of Units indicating that they had stratified such trials by treatment provider only (*n* = 3/16, 19%).

CTUs with experience running trials in Scenario D, i.e. trials recruiting from multiple centres, each with multiple treatment providers and investigating a surgical intervention (*n* = 25/44, 57%), also primarily stratified by centre only (*n* = 21/25, 84%). One-fifth indicated stratifying by both centre and treatment provider in such trials (*n* = 5/25, 20%).

Whilst Units had less experience running trials like Scenario E, which was similar to Scenario D but investigating substantially different interventions, stratification approaches were similar to those of Scenario D (centre only: 13/16, 81%; both centre and treatment provider: 2/16, 13%).

Twelve responders provided free text explaining their approaches for stratification in each of the scenarios (Question 3*,* Supplementary Table 5). Two-thirds (*n* = 8/12, 67%) commented on the feasibility of stratifying by treatment provider. Reasons were as follows: concerns that there would be too few per strata [ID8, ID15, ID39]; treatment provider not known in advance [ID8, ID32]; delivered by a subset of treatment deliverers [ID1, ID39]; data not collected on treatment provider [ID13]; treatment differences assumed to be differences in facilities and protocols [ID17]; usually comparing the intervention policy and not the different aspects of the intervention [ID32]; treatment provider can change during the trial [ID30].

Other responses provided examples of stratification levels, e.g. centre as hospital and treatment provider as operating surgeon [ID10]; two that this was trial-specific [ID14, ID29]. One raised concerns with stratifying by centre:

“*Recent conversions between senior statisticians advocate not stratifying by centre in any situation. They cited concerns regarding prediction of allocation.*” [ID18]

When comparing stratification approaches across scenarios within Units (Question 3, Table 2), 19 Units used the same approach across all scenarios in which they had experience, and 20 changed their approach depending on the trial scenario (same: *n* = 19/44, 43%; different: *n* = 20/44, 46%). Five had no experience in any of the suggested scenarios or did not respond to the question.

#### Learning

The majority of responders (*n* = 39/44, 89%) indicated they had accounted for learning by defining a minimum level of expertise for treatment providers (Question 4, Table 2). Common definitions were set in terms of delivering the trial intervention (*n* = 31/44, 70%); treating the condition within the patient population (*n* = 24/44, 55%); and setting a minimum professional level for treatment providers (*n* = 22/44, 50%). Three delegated this responsibility to the clinical investigators on the study. Examples of alternative approaches to specifying minimum levels of expertise included use of a surgical manual with senior surgeons signing off treatment deliverers [ID15] and treatment deliverers being required to pass both surgical and radiotherapy quality assurance [ID18].

Thirty percent of CTUs had used an expertise-based trial design, in which participating treatment providers provide only the intervention in which they have expertise (*n* = 13/44, 30%, Question 5, Table 2).

### Managing effects through analysis

#### Clustering

In trials stratified by centre, 55% of Units had subsequently adjusted by this stratification factor in the analysis (*n* = 24/44, 55%, Question 6, Tables 4 and 5). This had been done either by pre-specified grouping rules at the design stage (*n* = 19/24, 80%); by an ad hoc approach (*n* = 14/24, 58%); or by other approaches: grouped centres where numbers are small [ID7, ID15]; site as a fixed effect [ID8]; or:

“*Depends. Either include as a stratifying factor (small number of centres, large patient numbers) or by including centre or treatment provider as a cluster*.” [ID32]

**Table 4:** Methods for managing clustering and learning by analysis

Question			Category	Response statistics		
				*n*	*N*	*n*/*N*%
6	a	Assuming that you have stratified by centre, do you combine by the stratification factor for the purpose of analysis? If so how? *See Table**5**for further details.*	Yes	24	44	55%
			Pre-specified grouping rules at design stage	19	24	80%
			Ad hoc approach, e.g. determined after design due to small numbers per group	14	24	58%
			Other grouping rule or further details provided	6	24	26%
			No	17	44	39%
			No response	3	44	7%
	b	Assuming that you have stratified by treatment provider, do you combine by the stratification factor for the purpose of analysis? If so how?	Yes	16	44	36%
			Pre-specified grouping rules at design stage	12	16	75%
			Ad hoc approach, e.g. determined after design due to small numbers per group	7	16	44%
		*See Table**5**for further details.*	Other grouping rule or further details provided	5	16	31%
			No	14	44	32%
			No experience with trials of this type	1	44	2%
			No response	13	44	30%
7	Does your Unit include centre in the statistical model when comparing treatment?		Yes	39	44	89%
			But only if it was used to stratify randomisation	18	39	46%
			Always	6	39	15%
			Sometimes^a^	15	39	38%
			No, never	3	44	7%
			No response^b^	2	44	5%
	a	If yes, and assuming that the sample size allows either, would you treat this effect as fixed or random? *See Supplementary Box**3**for further details.*	Fixed or random, depending on circumstances	14	39	36%
			Fixed	11	39	28%
			Random	12	39	31%
			No response	2	39	5%
8	Does your Unit include treatment provider in the statistical model when comparing treatment?		Yes	26	44	59%
			But only if it was used to stratify randomisation	8	26	31%
	*See Supplementary Box**4**for further details.*		Always	0	26	0%
			Sometimes^c^	18	26	69%
			No, never	13	46	30%
			No response^d^	5	44	11%
	a	If yes, and assuming that the sample size allows either, would you treat this effect as fixed or random?	Fixed or random, depending on circumstances	4	26	15%
			Fixed	2	26	8%
			Random	18	26	69%
			No response	2	26	8%
	b	If yes, has this effect ever been treated as time-varying within the statistical model?	Yes	2	26	8%
			No	21	26	81%
			No response	3	26	12%
9	In each of the following scenarios, regardless of the randomisation stratification approach, has a treatment by centre or surgeon interaction investigated, in trials that your Unit has run? Select all that apply. *See Supplementary Table**6**for further details.*					
	A	Large sample size,^e^ recruiting in several centres, each with multiple treatment providers	Experience in trial type	35	44	80%
			Centre	16	35	46%
			Treatment provider	4	35	11%
			Both	3	35	9%
			Neither	20	35	57%
			No experience in trial type	7	44	16%
			No response	2	44	5%
	B	Small sample size,^f^ With centres each recruiting 2 to 3 patients	Experience in trial type	30	44	68%
			Centre	5	30	17%
			Treatment provider	0	30	0%
			Both	0	30	0%
			Neither	25	30	83%
			No experience in trial type	12	44	27%
			No response	2	44	5%
	C	Recruiting in several centres, where treatment providers treat patients across recruiting centres (treatment provider is not unique to a centre)	Experience in trial type	15	44	34%
			Centre	4	15	27%
			Treatment provider	1	15	7%
			Both	0	15	0%
			Neither	11	15	73%
			No experience in trial type	27	44	61%
			No response	2	44	5%
	D	A trial investigating a surgical intervention, recruiting from several centres, each with multiple treatment providers	Experience in trial type	21	44	48%
			Centre	5	19	24%
			Treatment provider	3	19	14%
			Both	1	19	5%
			Neither	14	19	67%
			No experience in trial type	19	44	43%
			No response	4	44	9%
	E	Recruiting from several centres, each with multiple treatment providers, comparing substantially different interventions, e.g. surgery to an injection	Experience in trial type	14	44	32%
			Centre	5	14	36%
			Treatment provider	1	14	7%
			Both	0	14	0%
			Neither	9	14	64%
			No experience in trial type	26	44	59%
			No response	4	44	9%
	In scenarios where Unit has experience, approaches to stratification changes across scenario, i.e. within-Unit variation to stratification		Different approaches across scenarios	12	44	27%
			Same approach across all scenarios	24	44	55%
			No response to Question 9	8	44	18%
10	a	If a positive treatment effect is found, does your Unit explore heterogeneity of treatment effects by centre? *See Supplementary Table**7**for further details.*	Yes	32	44	73%
			No	9	44	20%
			No response	3	44	7%
		i. If yes to a, do you explore by graphical display?	Yes	31	32	97%
			No	0	32	3%
			No response	1	32	3%
		ii. If yes to a, do you explore by analytical methods, e.g. significance testing?	Yes	22	32	69%
			No	5	32	16%
			No response	5	32	16%
	b	If a positive treatment effect is found, does your Unit explore heterogeneity of treatment effects by treatment provider? *See Supplementary Table**8**for further details.*	Yes	12	44	27%
			No	23	44	52%
			No response	9	44	20%
		i. If yes to b, do you explore by graphical display?	Yes	11	12	92%
			No	0	12	0%
			No response	1	12	8%
		ii. If yes to b, would you explore by analytical methods, e.g. significance testing?	Yes	9	12	75%
			No	1	12	8%
			No response	2	12	17%

^a^’Sometimes’ here is ’usually’—it is a rare exception where we don’t [ID10]

^b^No Standard Operating Procedure in place [ID3]

^c^’Sometimes’ here is ’usually’—it is a rare exception where we don’t [ID10]

^d^No experience in trials of this type [ID1]. Not applicable [ID2]

^e^With centres each recruiting at least 10 patients

^f^With centres each recruiting 2 to 3 patients

**Table 5:** Other grouping rules when randomisation is stratified by (a) centres or (b) treatment providers (Question 6)

*Centre stratified:*	
ID4	Would normally analyse together but adjust for stratification factors (which normally include centres) in analysis.
ID7	There will be instances where we have combined centres at the analysis stage due to small numbers.
ID8	Different statisticians/trials do different things. Often site = fixed effect and course within site = random effect. If too few within site, then would combine.
ID14	Retain structure at analysis.
ID15	Have grouped by region/country where numbers are small. Any adjustment should be documented in the Statistical Analysis Plan, and final decision regarding appropriateness can be discussed during blind review of data.
ID30	Have used both pre-specified and ad hoc approaches (due to recruitment issues).
*Not stratified by centre:*	
ID32	We either include as a stratification factor (small number of centres, large patient numbers) or by including centre/provider as a cluster.
*Treatment provider stratified:*	
ID7	Thinking about complex intervention studies, we don’t usually allow for a ’provider’ effect in the primary analyses, although not necessarily explicitly stated in protocol—many of these studies effectively have partial clustering. We’ve had recent interesting discussions regarding provider effect in such trials, with Chief Investigators strongly feeling that with standardised/manualised intervention and training, it isn’t relevant.
ID15	Any adjustment should be documented in the Statistical Analysis Plan, and final decision regarding appropriateness can be discussed during blind review of data.
ID24	Experience with multiple treatment providers is in oncology trials with different doctors delivering protocol treatment, e.g. chemotherapy/radiotherapy. The actual treating doctor has not been recorded on the Case Report Forms, hence all providers implicitly combined within a centre.
ID30	Have used both pre-specified and ad hoc approaches (due to recruitment issues).
ID39	Treatment providers combined by default—as we don’t routinely distinguish them in the analysis.

Regardless of the stratification approach used, very few Units had never adjusted for centre in the statistical model when comparing treatment (*n* = 3/44, 7%, Question 7, Table 4 and Supplementary Box 3). Responders from Units that did (39/44, 89%), did so using fixed effects (*n* = 11); random effects (*n* = 12); or, depending on the circumstance, used either (*n* = 14). Two did not respond. Reasons in favour of using fixed effects were ease of interpretation and fewer assumptions associated with it [ID27]; and random effects as:

*“Usually an underlying assumption that centre may be a surrogate for socioeconomic factors that may affect outcome and/or treatment effect and so often not happy to assume that there is an equal fixed treatment effect across all sites.*” [ID15]

In trials stratified by treatment provider, 36% also subsequently adjusted the analysis (*n* = 16/44, 36%, Question 6, Tables 4 and 5). Three-quarters did so in accordance with pre-specified grouping rules (*n* = 12/16, 75%) or using a more ad hoc approach (*n* = 7/16, 44%).

Regardless of stratification approach used, 59% adjust for treatment provider in the statistical model when comparing treatment (*n* = 26/44, 59%, Question 8, Table 4 and Supplementary Box 4). The majority of responders used a random effect (*n* = 18/26, 69%), with one providing reason:

*“If treatment provider was included as stratification factor it will be because we are concerned that the provider will have an impact on outcome but also because we would expect different population for different treatment providers.”* [ID15]

When responders were asked to revisit the scenarios in Table 1*,* this time to consider investigating treatment by centre or treatment provider (Question 9, Table 4), exploring treatment by centre was universally most common across all scenarios. Exploring treatment by provider was rare. Twelve responders provided free text to explain their approaches for adjustment (Question 9, Supplementary Table 6). General themes for additional information provided were as follows: that the decision is trial-dependent [ID6, ID14]; concerns around sample size [ID6, ID7, ID39]; and, when explored, that this was informal [ID5, ID8, ID14, ID32, ID38].

When comparing treatment interaction approaches across scenarios within Units (Question 9*,* Table 4), 24 Units used the same approach across all scenarios, and 12 utilised a scenario-specific approach (same: *n* = 24/44, 55%; different: *n* = 12/44, 27%). Eight had no experience in any of the suggested scenarios or did not respond to the question.

Seventy-three percent of Units explored heterogeneity by centre when a positive treatment effect is found (*n* = 32/44, 73%, Question 10a, Table 4), whereas fewer explored heterogeneity by treatment provider (*n* = 12/44, 27%, Question 10b, Table 4). Of those that do explore heterogeneity for either effect, the majority did so by graphical display (centre: *n* = 31/32; treatment provider: *n* = 11/12). Many also explored by analytical methods, for example significance testing (centre: *n* = 22/32; treatment provider: *n* = 9/12). Supplementary Tables 6 and 7 provide further details.

#### Learning

Fifty-nine percent of CTUs included the treatment provider in the statistical model when comparing treatment (*n* = 26/44, 59%), two of which had treated this as a time-varying covariate (Question 8, Table 4), with one specifying:

*“Fairly crude by letting the number of procedures in the trial increase the relevant surgeon’s experience (ignoring procedures done outside of the trial of course!)”* [ID38]

Those that had not used a time-varying effect had experience in exploring learning through a sensitivity analysis [ID35] or secondary analyses [ID8, ID39], with one specifying:

*“Had we found evidence of learning, we would have had awkward additional data summaries and presentations”* [ID8]

Two responders had not considered such analyses [ID7, ID23], and one provided time restrictions as a reason for not doing so [ID30].

## Discussion

This survey identifies the fact that, despite multicentre trials being prominent across all CTUs, there is a UK-wide variation of designing and analysing these trials with respect to clustering and learning effects. Approximately half of Units changed their approach to design and analysis when presented with five example trial scenarios, each with varying levels of complexity, such as small sample size per centre and complex interventions, such as surgery. This finding suggests that variation can exist both across and within Units, suggesting that this decision can depend on the type of trial being conducted. Units indicate awareness of the potential methodological challenges associated with the design and analysis of multicentre trials, although the approaches used and opinions on these vary. The high response rate achieved provides insight into the general and current practice of managing clustering and learning effects in multicentre trials investigating varying types of interventions. Whilst acknowledging that different approaches may be more suitable to different trial types, they indicate the need for a more unified approach to the design and analysis of trials where outcomes are associated with the delivery of the intervention and/or more research in this field.

When adjusting for clustering within the design, a higher proportion than expected ran trials that did not stratify by centre (52%). Most commonly, this was due to too many centres and not enough participants within centre. Stratifying by centre was most common in all scenarios, whilst stratifying by treatment provider was consistently rare but more common in trials with a surgical intervention. Stratifying by treatment provider raised pragmatic concerns, e.g. concerns over relevance to research question, or provider not known pre-randomisation. Whilst in some settings, such as emergency treatment, advance knowledge of the treatment provider will be unobtainable, advanced planning may be possible in other settings, such as group therapy, with guidance for practical issues like these available [13]. Half of the responders had adjusted by centre following stratification by the same. Most commonly this was done by pre-specified grouping rules established at the design stage or using an ad hoc approach determined after design due to small numbers per group. Regardless of stratification approach, eight-tenths of responders had adjusted for centre in the statistical model. There were mixed opinions on how this adjustment was made, i.e. by fixed or random effects with reasons provided for and against both approaches. When a positive treatment effect is found, three-quarters and one-third stated that they then explore heterogeneity by centre and treatment provider respectively. All did so using graphical displays.

Managing learning by design through defining a minimum level of expertise for health professionals participating in the trial [4] was most common, with almost all responders (89%) applying this approach to studies within their Unit. Less than a third indicated experience in conducting expertise-based designs, a design that can be particularly useful when comparing substantially different interventions. This finding suggests these designs are more commonly implemented than suggested by the literature [7, 14]. Concerns were raised that identifying evidence of learning may lead to ’awkward additional data summaries’.

Guidance on trial design and analysis does exist, with the most relevant of these recommendations being explicitly incorporated into the survey questions [4, 6] Supplementary Box 2. Additional documents within the International Council for Harmonisation (ICH) Series provide further guidance beyond ICH E9 [15, 16]. The Consolidated Standards of Reporting Trials (CONSORT) statement and relevant extensions provide direction valuable at study design despite the document being developed to support reporting [17, 18]. The decision to explore effects may, in part, be related to the intention of the research in terms of how the results will be used, and the PRECIS-2 tool has been developed to help with this [19]. However, the ability to identify and explore heterogeneity at the analysis stage is an important consideration for generalisability for all trials.

Strengths of this study were that, although the survey was limited to registered Units, responders represent wide geographic coverage within the UK, spanning a diverse range of medical conditions and associated methodologies. In addition, participating Units are known to comply with required regulatory standards and meet acceptable standards of quality required by the UKCRC CTU registration process [20]. All responders were experienced triallists who either were statistical lead at their Unit or a nominated statistical representative. Publicly funded trials cover a diversity of interventions [15] and are generally not seeking a marketing authorisation from the competent authorities, and this may impact the approaches taken in line with heterogeneity of effects by cluster or time. Limitations of this work are that it represents statistical practice within the UK in leading trial centres, with global practice unknown. However, the survey drew upon internationally accepted guidelines [4] for best practice, and therefore the opinions and experiences are applicable beyond the UK. Second, some of the observed responses may have related to the different types of trials that the CTUs conduct. Not all trials include interventions where there is learning. Indeed, one would anticipate that many pragmatic large-scale trials do not have ‘learning’ effects because they include interventions that are stabilised and in widespread use. Whilst the survey allowed for free text responses, a more focussed survey, achieved using qualitative research methods, would be needed to examine these issues. Third, the volume of studies designed by each Unit will vary widely, and one responder per Unit may result in experiences reported for larger Units not being indicative of all studies run. However, responders were able to complete the survey with additional support within their Unit if deemed appropriate.

## Conclusions

This survey is the first to report on the experience and management approaches with regard to clustering effects and the learning curve in multicentre randomised trials. Importantly, responders, who were highly experienced in the design and analysis of such studies, appear to have awareness of when to make such considerations. Whilst approaches to management are varied, and this variation may be trial-dependent within Unit, reasons for approaches reported were provided and approaches justified. Historically, guidance on the design, analysis and reporting of randomised controlled trials was developed more generally to support consistency in approaches across a more conventional randomised controlled trial [4, 15, 16], with the development of more intervention-specific guidelines being established following these to address the additional complexities across different types of trials [11, 17, 18, 21]. Intervention-specific guidelines may have led to the variation and justifications identified in this survey. Results highlight the need for better consistency between triallists. Agreeing principles to guide trial design and analysis across a range of realistic clinical scenarios should be considered and/or further researched to establish optimal methods.

## Supplementary information

**Additional file 1: Supplementary Box 1.** List of UK Clinical Research Collaboration Registered Clinical Trials Units.

**Additional file 2: Supplementary Box 2.** Survey.

**Additional file 3: Supplementary Table 1.** CTU completion rate.

**Additional file 4: Supplementary Table 2.** Role of Unit representative.

**Additional file 5: Supplementary Table 3.** CTU completion rates by specialty trial types.

**Additional file 6: Supplementary Table 4.** Experience in running complex and/or surgical interventions.

**Additional file 7: Supplementary Table 5.** Comments on stratification approaches in example scenarios (Question 3).

**Additional file 8: Supplementary Box 3.** Reasons for using fixed or random effect for centre (Question 7).

**Additional file 9: Supplementary Box 4.** Reasons for using fixed, random or time-varying effect for treatment provider (Question 8)

**Additional file 10: Supplementary Table 6.** Comments on interaction investigation in example scenarios (Question 9).

**Additional file 11: Supplementary Table 7.** Details of how Units explore heterogeneity by centre in the presence of a treatment effect (Question 10a).

**Additional file 12: Supplementary Table 8.** Details of how Units explore heterogeneity by treatment provider in the presence of a treatment effect (Question 10b).

## Data Availability

The anonymised datasets used and/or analysed during the current study are available from the corresponding author on reasonable request.
