# Supplementary material for: Managing clustering effects and learning effects in the design and analysis of multicentre randomised trials: a survey to establish current practice
Source: Trials. 2020 May 27;21:433. doi: 10.1186/s13063-020-04318-x (PMC7251810; doi:10.1186/s13063-020-04318-x)
Supplement: Supplementary file 1 — Additional file 1: Supplementary Box 1. List of UK Clinical Research Collaboration Registered Clinical Trials Units. [file 13063_2020_4318_MOESM1_ESM.docx]

**Supplementary Box 1: List of UK Clinical Research Collaborative Registered Clinical Trials Units**

The following list of 51 Units was obtained on 4^th^ January 2019:

Barts and the London Pragmatic CTU

Barts Clinical Trials Unit

Birmingham Clinical Trials Unit

Brighton and Sussex Clinical Trials Unit

Bristol Clinical Trials and Evaluation Unit

Bristol Randomised Trials Collaboration

CaCTUS (Cancer Clinical Trials Unit Scotland)

Cambridge Clinical Trials Unit (CCTU)

Cambridge Epidemiology & Trials Unit

Cancer Research UK Clinical Trials Unit (CRCTU)

Centre for Healthcare Randomised Trials (CHaRT)

Centre for Trials Research

Comprehensive CTU @ UCL

CR UK & UCL Cancer Trials Centre

Derby Clinical Trials Support Unit (DCTSU)

Diabetes Trials Unit (Churchill Hospital, Oxford)

Edinburgh Clinical Trials Unit, Edinburgh

Exeter Clinical Trials Unit

Glasgow Clinical Trials Unit

Imperial Clinical Trials Unit

Intensive Care National Audit & Research Centre (ICNARC) CTU

Keele Clinical Trials Unit

King's Clinical Trials Unit at King's Health Partners

Leeds Clinical Trials Research Unit

Leicester Clinical Trials Unit

Liverpool Trials Collaborative

London School of Hygiene & Tropical Medicine

Manchester Academic Health Science Centre Clinical Trials Unit (MAHSC-CTU)

Medical Research Council Clinical Trials Unit at UCL

Newcastle Clinical Trials Unit (NCTU)

NHS Blood and Transplant Clinical Trials Unit

North Wales Organisation for Randomised Trials in Health (NWORTH)

Northern Ireland Clinical Trials Unit

Norwich Clinical Trials Unit

Nottingham Clinical Trials Unit

NPEU Clinical Trials Unit

Oxford Clinical Trial Service Unit & Epidemiological Studies Unit (CTSU)

Oxford Clinical Trials Research Unit (OCTRU)

Oxford Primary Care and Vaccines Collaborative Clinical Trials Unit

Papworth Trials Unit Collaboration

Peninsula Clinical Trials Unit

PRIMENT Clinical Trials Unit at UCL

Royal Marsden Clinical Trials Unit (RM-CTU)

Sheffield Clinical Trials Research Unit

Southampton Clinical Trials Unit

Surrey Clinical Trials Unit

Swansea Trials Unit

Tayside Clinical Trials Unit

The Institute of Cancer Research Clinical Trials & Statistics Unit (ICR- CTSU)

Warwick Clinical Trials Unit

York Trials Unit
