# Supplementary figures and images for: Managing clustering effects and learning effects in the design and analysis of multicentre randomised trials: a survey to establish current practice
Source: Trials. 2020 May 27;21:433. doi: 10.1186/s13063-020-04318-x (PMC7251810; doi:10.1186/s13063-020-04318-x)

**Supplementary Box 2: Survey**
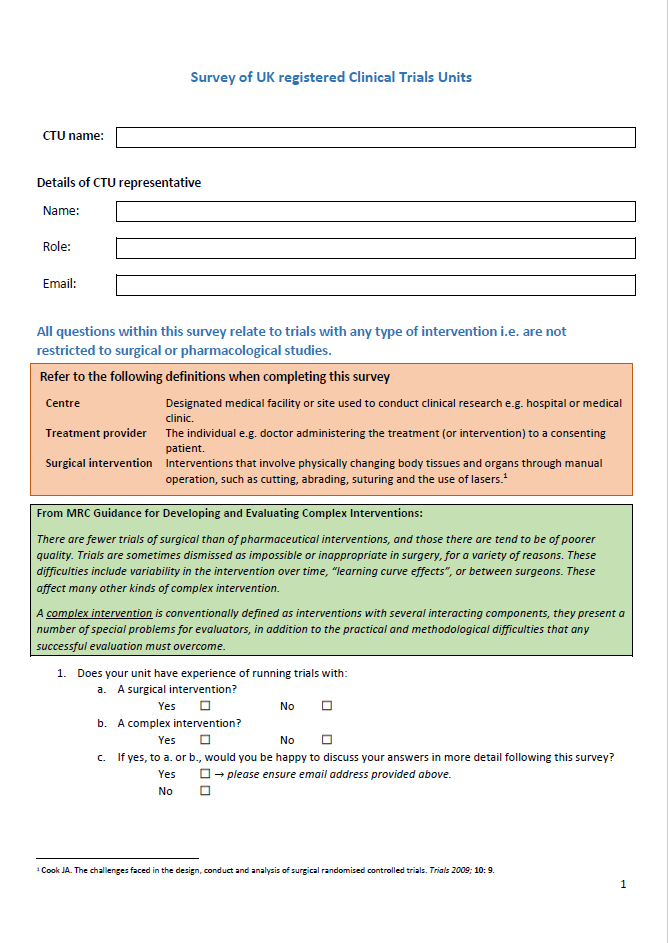


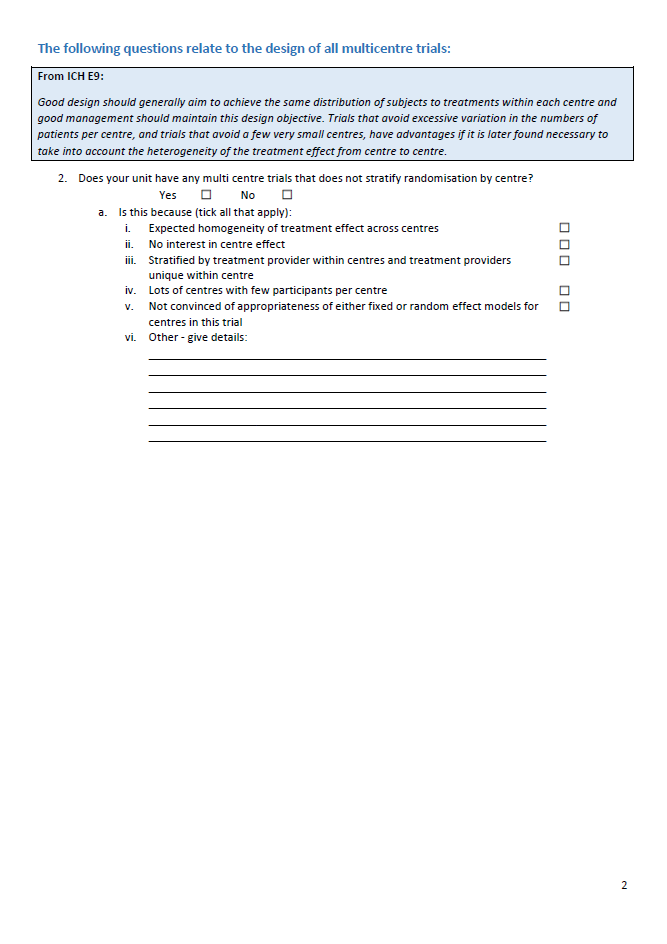


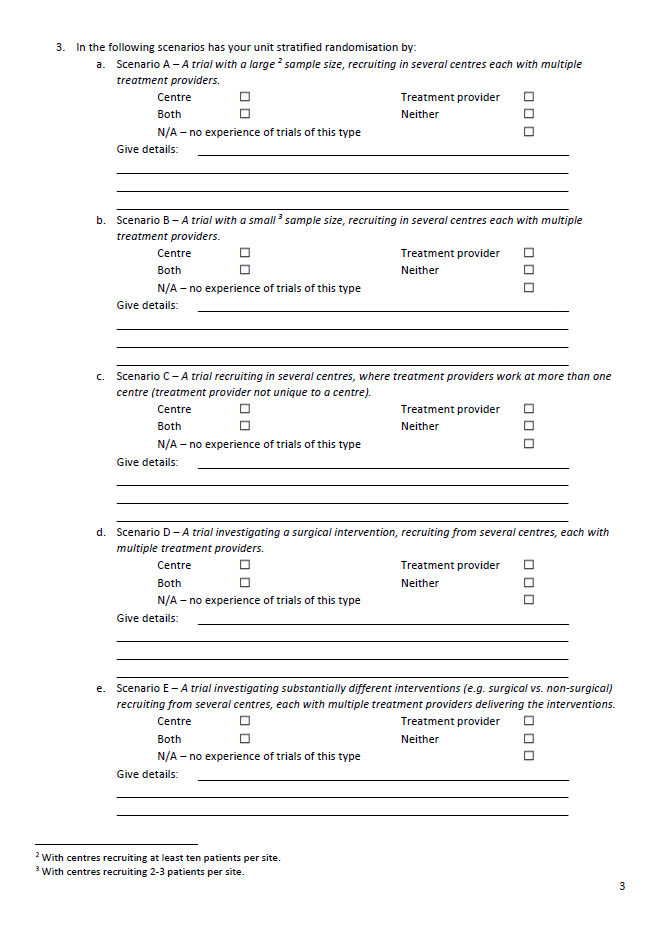


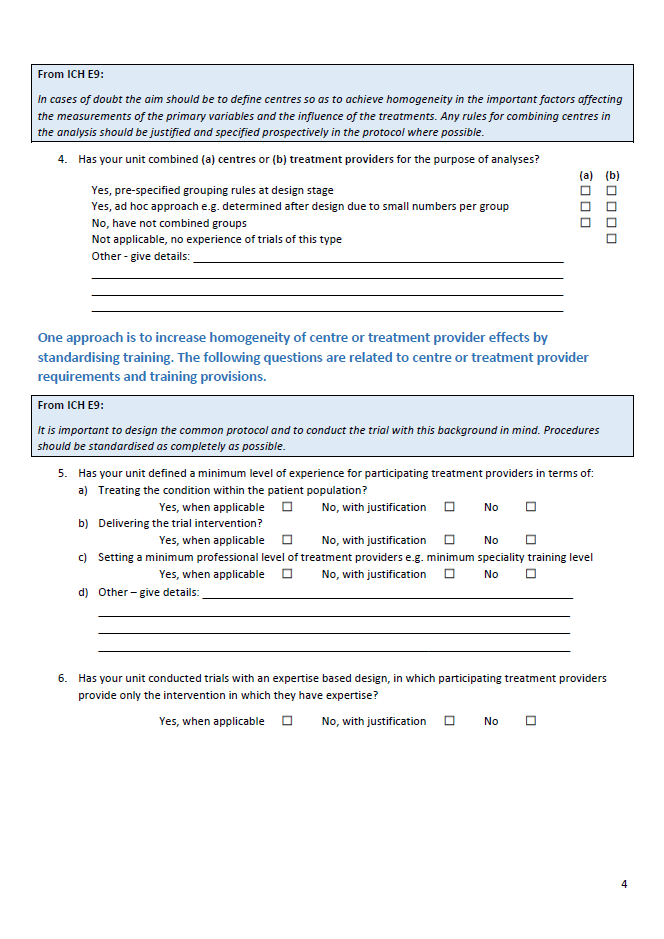


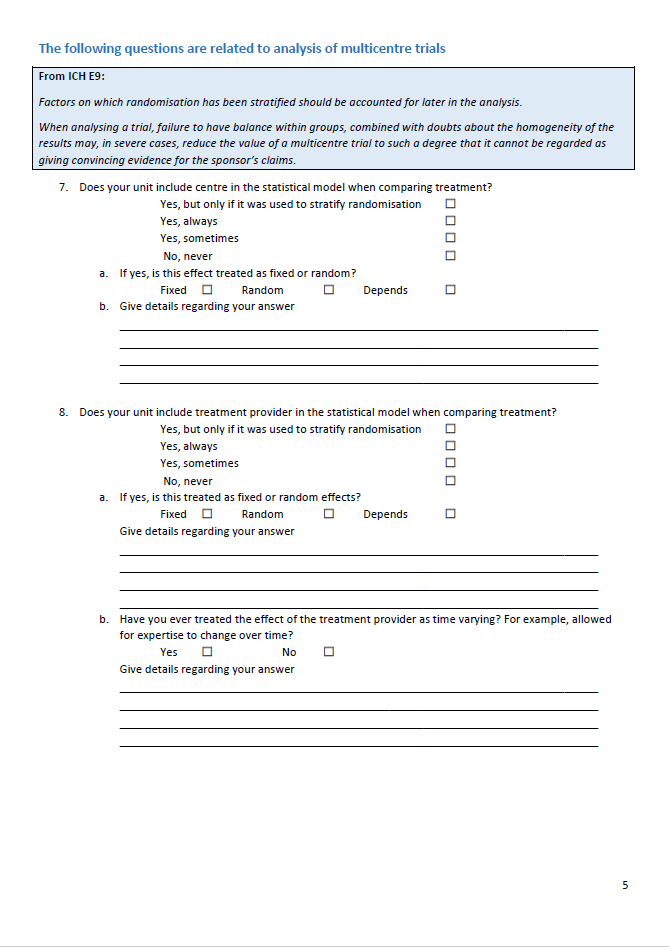


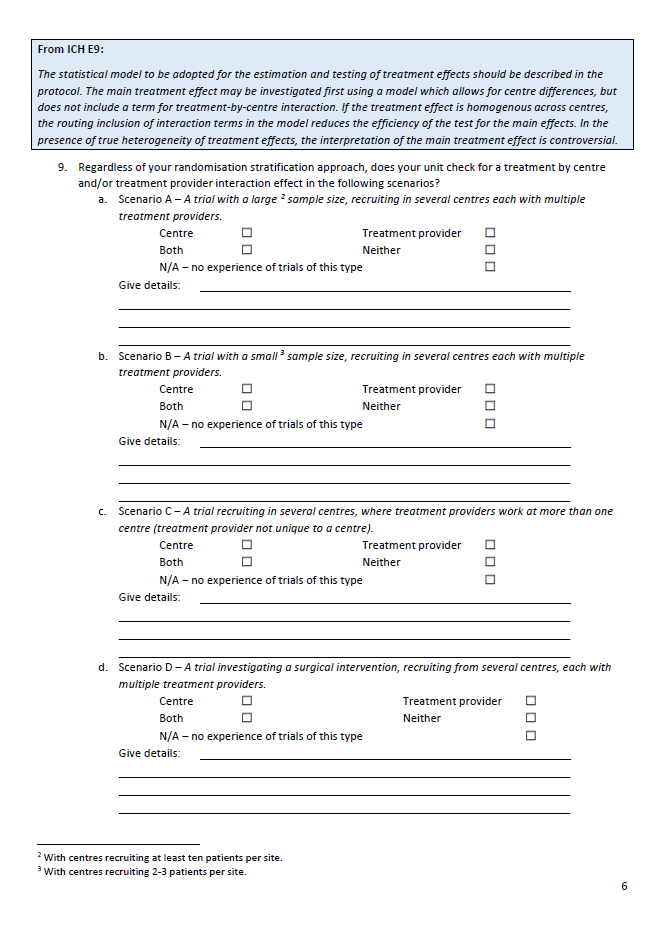


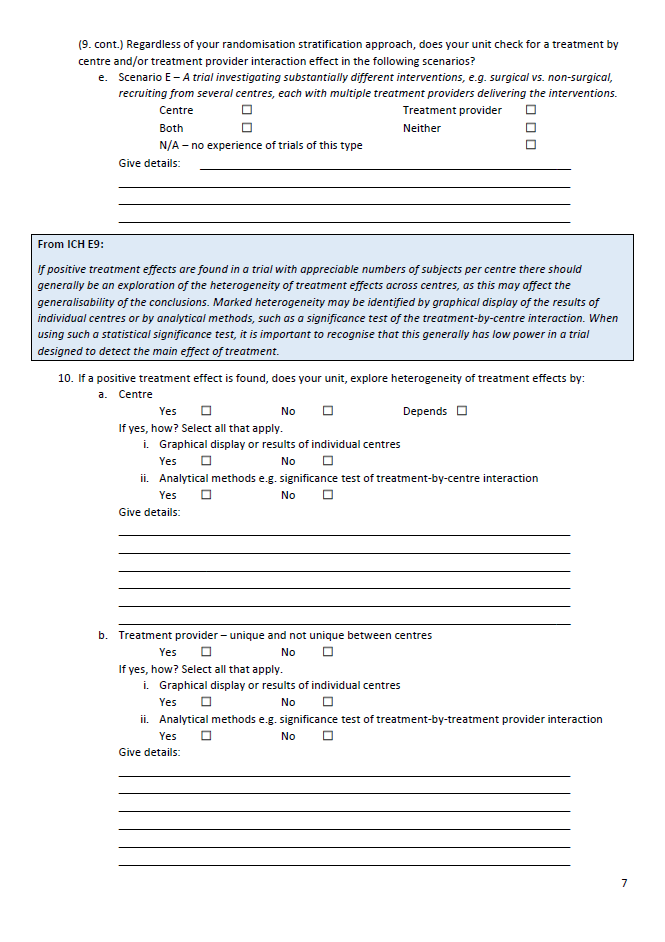


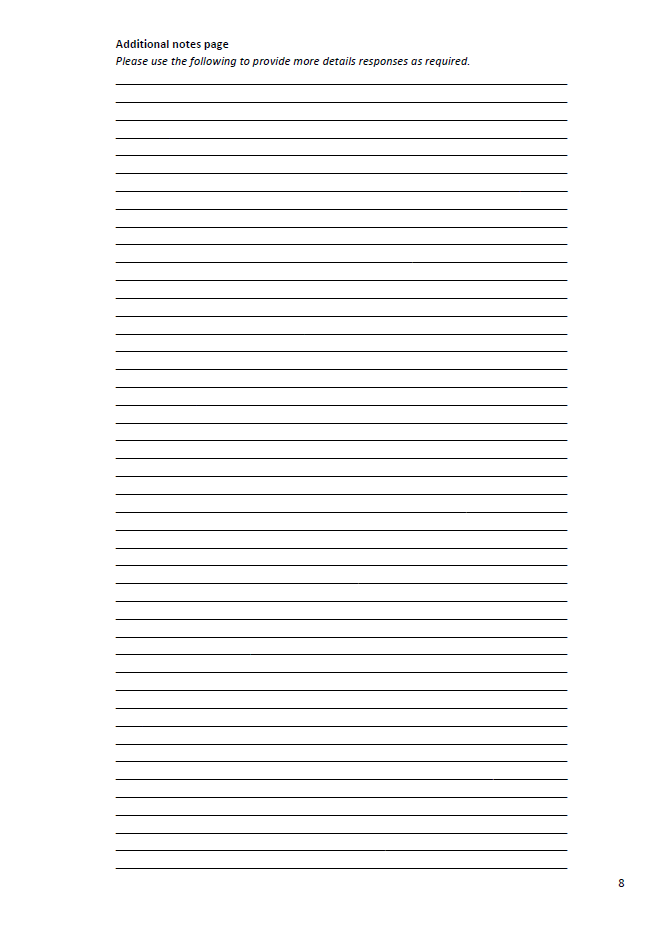

Supplement: Supplementary file 2 — Additional file 2: Supplementary Box 2. Survey. [file 13063_2020_4318_MOESM2_ESM.docx]
