# Supplementary material for: Managing clustering effects and learning effects in the design and analysis of multicentre randomised trials: a survey to establish current practice
Source: Trials. 2020 May 27;21:433. doi: 10.1186/s13063-020-04318-x (PMC7251810; doi:10.1186/s13063-020-04318-x)
Supplement: Supplementary file 3 — Additional file 3: Supplementary Table 1. CTU completion rate. [file 13063_2020_4318_MOESM3_ESM.docx]

**Supplementary Table 1: CTU completion rate**

| Participation status | Number of Units | | |
| --- | --- | --- | --- |
|  |  |  |  |
|  | n | N | n/N% |
| Completed | 44 | 50 | 88% |
| Pen and paper | 31 | 44 | 70% |
| Electronic form | 13 | 44 | 30% |
| Declined, unable to participate | 1 | 50 | 2% |
| Declined, no reason provided | 5 | 50 | 10% |
