# Supplementary material for: Managing clustering effects and learning effects in the design and analysis of multicentre randomised trials: a survey to establish current practice
Source: Trials. 2020 May 27;21:433. doi: 10.1186/s13063-020-04318-x (PMC7251810; doi:10.1186/s13063-020-04318-x)
Supplement: Supplementary file 4 — Additional file 4: Supplementary Table 2. Role of Unit representative. [file 13063_2020_4318_MOESM4_ESM.docx]

**Supplementary Table 2: Role of Unit representative**

| Role | Number of Units | | |
| --- | --- | --- | --- |
|  |  |  |  |
|  | n | N | n/N% |
| Professor/Reader of Medical Statistics | 3 | 44 | 7% |
| Director of Unit | 6 | 44 | 14% |
| Statistics lead/Head of statistics | 13 | 44 | 30% |
| Senior statistician | 15 | 44 | 34% |
| Statistician | 7 | 44 | 16% |
