# Supplementary material for: Managing clustering effects and learning effects in the design and analysis of multicentre randomised trials: a survey to establish current practice
Source: Trials. 2020 May 27;21:433. doi: 10.1186/s13063-020-04318-x (PMC7251810; doi:10.1186/s13063-020-04318-x)
Supplement: Supplementary file 5 — Additional file 5: Supplementary Table 3. CTU completion rates by specialty trial types. [file 13063_2020_4318_MOESM5_ESM.docx]

**Supplementary Table 3: CTU completion rates by speciality trial types**

|  |  | Specialises in trial of type | | | | | | | | | | | |
| --- | --- | --- | --- | --- | --- | --- | --- | --- | --- | --- | --- | --- | --- |
|  |  | Complex intervention | | | | Surgical intervention | | | | Cluster randomised | | | |
| Completer status | | Yes  N=39 | | No  N=11 | | Yes  N=36 | | No  N=14 | | Yes  N=17 | | No  N=33 | |
|  |  | N | n/N% | N | n/N% | n | n/N% | n | n/N% | n | n/N% | n | n/N% |
| Completed | | 35 | 90% | 9 | 82% | 33 | 92% | 11 | 79% | 16 | 94% | 28 | 85% |
| Declined, unable to participate | | 1 | 3% | 0 | 0% | 1 | 3% | 0 | 0% | 1 | 6% | 0 | 0% |
| Declined, no reason provided | | 3 | 8% | 2 | 18% | 2 | 6% | 3 | 21% | 0 | 0% | 5 | 15% |
