# Supplementary material for: Managing clustering effects and learning effects in the design and analysis of multicentre randomised trials: a survey to establish current practice
Source: Trials. 2020 May 27;21:433. doi: 10.1186/s13063-020-04318-x (PMC7251810; doi:10.1186/s13063-020-04318-x)
Supplement: Supplementary file 6 — Additional file 6: Supplementary Table 4. Experience in running complex and/or surgical interventions. [file 13063_2020_4318_MOESM6_ESM.docx]

**Supplementary Table 4: Experience in running complex and/or surgical interventions**

|  | | |  | Response statistics | | |
| --- | --- | --- | --- | --- | --- | --- |
| Question | | | Category | n | N | n/N% |
| 1 | Which of the following intervention trials does your Unit have experience of running | | Both surgical and complex interventions | 25 | 44 | 57% |
|  |  |  | Surgical interventions only | 4 | 44 | 9% |
|  |  |  | Complex interventions only | 7 | 44 | 16% |
|  |  |  | Neither | 7 | 44 | 16% |
|  |  |  | No response | 1 | 44 | 2% |
