# Supplementary material for: Managing clustering effects and learning effects in the design and analysis of multicentre randomised trials: a survey to establish current practice
Source: Trials. 2020 May 27;21:433. doi: 10.1186/s13063-020-04318-x (PMC7251810; doi:10.1186/s13063-020-04318-x)
Supplement: Supplementary file 7 — Additional file 7: Supplementary Table 5. Comments on stratification approaches in example scenarios (Question 3). [file 13063_2020_4318_MOESM7_ESM.docx]

**Supplementary Table 5: Comments on stratification approaches in example scenarios (Question 3)**

| ID | Scenario | Stratification approaches | Free text |
| --- | --- | --- | --- |
| ID1 | A | No experience | For A, our trials are in a critical care setting – this is a team based specialty and it is generally not possible to distinguish individual treatment providers |
|  | B | No experience |  |
|  | C | No experience |  |
| ID3 | A | No experience | Have not run studies with several centres. A study with two sites that we run stratifies randomisation by neither centre nor treatment provider. |
|  | B | No experience |  |
|  | C | No experience |  |
| ID4 | A | Centre only  Both | For A, most commonly just by centre |
|  | B | No experience |  |
|  | C | Centre only |  |
| ID8 | A | Centre only | I do not think we have any behavioural therapy/course type interventions at our Unit, but stratifying by site in these seems OK. If you were to stratifying by deliverer, there is likely to be too few per strata. For cross nesting, this is often delivered by >1 treatment provider. There is a group effect or course effect rather than therapy effect in this case. For example, personalities or dynamics. I do not see this changing unless convinced otherwise. |
|  | B | Centre only |  |
|  | C | Centre only |  |
|  | D | Centre only | Responses based on one trial. Not always known who surgeon will be in advance and too few per strata as often one surgeon. But here, I could be convinced. Note: In previous trials that I have analysed (1 surgery, 2 group) observed ICC=0. Again, anecdotal but I am unconvinced we need to change. |
|  | E | No experience |  |
| ID10 | A | Centre only | For A, centre: ambulance station. For B, feasibility: centre: ambulance station – provider: paramedic. |
|  | B | Centre only  Both |  |
|  | C | No experience |  |
|  | D | Centre only  Treatment provider only  Both | For D, centre: Hospital; Treatment provider: operating surgeon, closing the wound. |
|  | E | No experience |  |
| ID13 | A | Centre only | We rarely collect data on treatment provider. |
|  | B | Centre only |  |
|  | C | No experience |  |
| ID14 | A | Centre only  Both | For a, dependent on trial. General comments - considered and decided on a trial by trial basis. Don't feel you can always standardise. |
|  | B | Centre only |  |
|  | C | Treatment provider only |  |
|  | D | Centre only  Treatment provider only | General comments - considered and decided on a trial by trial basis. Don't feel you can always standardise. |
|  | E | Centre only |  |
| ID15 | A | Centre only  Treatment provider only  Both | We would generally stratify by centre as a rule of thumb if we’re happy we'll get enough patients per site. Stratifying by treatment provider is only carried out currently on the larger surgical studies = again where we're confident that there will be enough patients. Also depends on what other stratification factors are needed. |
|  | B | Centre only |  |
|  | C | Centre only |  |
| ID17 | A | Centre only | It is assumed that differences in treatment will mainly be due to differences in facilities in each centre and different treatment protocols within each centre. |
|  | B | Centre only |  |
|  | C | Centre only |  |
| ID18 | A | Centre only  Neither centre nor treatment provider | Recent conversions between senior statisticians advocate not stratifying by centre in any situation. They cited concerns regarding prediction of allocation. |
|  | B | Neither centre nor treatment provider |  |
|  | C | No experience |  |
|  | D | No experience | For D and E, not aware of any locally, but if we did then think definitely by treatment provider. |
|  | E | No experience |  |
| ID29 | A | Both | For C, stratification is likely to be chosen by the expected homogeneity, so may be centre or treatment provider. This will be intervention specific. |
|  | B | Centre only |  |
|  | C | Centre only  Treatment provider only |  |
| ID30 | A | Centre only | Only centre used as treatment provider could vary during the trial. This would add logistics of then having to update the randomisation protocol. |
|  | B | No experience |  |
|  | C | No experience |  |
| ID32 | A | Centre only | We often include treatment providers as a cluster effect but do not usually stratify as do not always know at randomisation. For c specifically, centres combined out of necessity. |
|  | B | Centre only |  |
|  | C | Centre only |  |
|  | D | Centre only | For D and E, usually comparing the intervention policy not the different aspects of the intervention. We try to standardise the interventions to make them as similar as possible. We have done surgery vs. physiotherapy for example. |
|  | E | Centre only |  |
| ID35 | A | Centre only | I can't remember every detail of every study on our books, I've written down my best guess. I don't know detail for other statistician's studies. |
|  | B | Neither centre nor treatment provider |  |
|  | C | Neither centre nor treatment provider |  |
| ID39 | A | Centre only | For A, Surgeons within seven centres. Surgery is conducted by a team which includes a variable subset of surgeons within a centre. Hence stratified by centre only, as would be unclear which surgeons to stratify by. For B, several pilot trials like this – to much stratification with a small sample size may not achieve balance across trial groups. |
|  | B | Centre only |  |
|  | C | No experience |  |
|  | D | Centre only | For D, Surgeons within seven centres. Surgery is conducted by a team which includes a variable subset of surgeons within a centre. Hence stratified by centre only, as would be unclear which surgeons to stratify by. For E, Surgery versus radiotherapy versus nurse-led active monitoring. Difficult to see how strata for randomisation could be defined at the practitioner level. |
|  | E | Centre only |  |
