# Supplementary material for: Managing clustering effects and learning effects in the design and analysis of multicentre randomised trials: a survey to establish current practice
Source: Trials. 2020 May 27;21:433. doi: 10.1186/s13063-020-04318-x (PMC7251810; doi:10.1186/s13063-020-04318-x)
Supplement: Supplementary file 8 — Additional file 8: Supplementary Box 3. Reasons for using fixed or random effect for centre (Question 7). [file 13063_2020_4318_MOESM8_ESM.docx]

**Supplementary Box 3: Reasons for using fixed or random effect for centre (Question 7)**

| *Use both fixed effect or random effect, as required (n=14):* | |
| --- | --- |
| ID1 | Always included in adjusted analyses, but sometimes a simple unadjusted analysis is also presented with no accounting for centre. Usually included as a random effect, except in studies with only a small number of centres e.g. pilot studies. |
| ID6 | Random if many centres, fixed if few. Also, some types of model don’t allow random effects e.g. quantile regression so no choice but to use fixed effects. |
| ID7 | If centre was used for stratification or minimisation then included in the primary statistical model. If centre wasn’t a stratification factor, on some occasions, centre might be included in exploratory/sensitivity analyses – partly depending on how many centres and sample size. The choice between fixed and random effects is trial specific – partly driven by whether the centres can be considered and/or justified as being a random sample and also the number of centres (low number would usually be modelled as fixed effects). |
| ID10 | Choice of fixed effects or random effects depends on number and/or nature of centres. |
| ID12 | Mainly random unless small number of centres. |
| ID14 | When a small number of centres, use fixed. |
| ID30 | Have treated as both but more likely to be random as this is more reflective of what we need. |
| ID35 | Random is >5 centres, fixed is lower number. (I think! Can’t remember the precise numeric cut off). Can’t do random effects very well if the number of centres is too low. |
| ID38 | Usually random, but if few centres (5 or less) I would use a fixed effect for centre. |
| ID39 | Centre is almost always included in the model – other than when a lot of centres have recruited only one or two patients e.g. primary care trials. Tends to be included as a fixed effect in a standard multi-centre trial, and a random effect in cluster randomised studies. |
| *Fixed effect for centre (n=11):* | |
| ID2 | Based on limited experience, centre was specified in the protocol as a fixed effect for one trial. |
| ID8 | Usually! I think it is easier to assess treatment by strata interactions in fixed effect. Debate is inevitable (even Stephen Senn says no firm views on this). |
| ID20 | Only one multivariate trial with four large centres and low heterogeneity between centres in the treatment effect. |
| ID27 | It requires fewer assumptions and easier to explain. If exact balance is achieved then the maths will give identical estimated standard errors with both models. |
| *Random effect for centre (n=12):* | |
| ID9 | We are interested in the impact of population of centres rather than the sample of centres used in the trial. |
| ID11 | Would aim for random effects but if not feasible (too few centres or too few per centre) then would include as a fixed effect. |
| ID13 | Uses fewer degrees of freedom if many centres and that we do not expect structured differences between centres. |
| ID15 | Usually an underlying assumption that centre may be a surrogate for socio-economic factors that may affect outcome and/or treatment effect so often not happy to assume that there is an equal fixed treatment effect across all sites. Would use random due to large number of centres. |
| ID23 | Depends a bit on the number of centres. Need to have a sufficient number to preserve the degrees of freedom. |
| ID29 | To report the centres as a sample of centres. |
| ID32 | Varies between statisticians – going more down the random effect moving forwards. |
