# Supplementary material for: Managing clustering effects and learning effects in the design and analysis of multicentre randomised trials: a survey to establish current practice
Source: Trials. 2020 May 27;21:433. doi: 10.1186/s13063-020-04318-x (PMC7251810; doi:10.1186/s13063-020-04318-x)
Supplement: Supplementary file 9 — Additional file 9: Supplementary Box 4. Reasons for using fixed, random or time-varying effect for treatment provider (Question 8) [file 13063_2020_4318_MOESM9_ESM.docx]

**Supplementary Box 4: Reasons for using fixed, random or time varying effect for treatment provider (Question 8)**

| *Use both fixed effect or random effect, as required (n=4):* | |
| --- | --- |
| ID7 | If treatment providers was used for stratification or minimisation then included in primary statistical model. For most of our complex intervention trials, we wouldn’t know who the provider is going to be at the time of randomisation and so can’t be a stratification/minimisation variable. We’ve considered e.g. partial clustering at the design stage where the intervention group has providers and the control group has treatment as usual, but usually insufficient information available, leading to potential for secondary/exploratory analyses of treatment provider. |
| ID35 | Depends on the number of providers. Can’t do random effects if number of providers is too low. Random is >5 centres and fixed is lower. I can’t think of an example where we have adjusted but we would if it were sensible. |
| *Fixed effect for treatment provider (n=2):* | |
| ID27 | It requires fewer assumptions and easier to explain. If exact balance is achieved then the maths will give identical estimated standard errors with both models. However, never actually done this. |
| ID30 | This is dependent on the data and requirements. |
| *Random effect for treatment provider (n=16):* | |
| ID8 | There are too few per strata in our trial to consider as fixed. May be qualitative assessment. |
| ID10 | Choice of random effect is based on parsimony. |
| ID15 | If treatment provider is included as a stratification factor, it’ll be because we’re concerned the provider will have an impact on the outcome but also because we’d expect different populations for different treatment providers. We have no interest in therapist effect for main adjustments, we would adjust to complement randomisation stratification factor. |
| ID23 | Depends on number, need to preserve degrees of freedom. |
| ID29 | To represent the result as a sample. |
| ID32 | As a cluster random effect. |
| ID39 | When a clear provider structure to the data, we will pre-specify the main analysis to accommodate this. We would try and capture the hierarchical structure in a multi-level model. |
| *Time-varying effect used for treatment provider (n=2):* | |
| ID38 | Fairly crude by letting the number of procedures in the trial increase the relevant surgeon’s experience (ignoring procedures done outside of the trial of course!) |
| *Time-varying effect not used for treatment provider (n=19):* | |
| ID7 | Interesting idea! Thinking again about our complex intervention trials, I’m not entirely sure that we would expect to see a change in treatment effect over time, as the Chief Investigator would, I suspect, say that with manualised interventions, etc, this shouldn’t be observed. But something to think about…. |
| ID8 | Secondary exploratory analyses. Had we found evidence of learning we would have had awkward additional data summaries/presentations. |
| ID10 | Not yet appropriate. |
| ID15 | Not yet appropriate in trials that we run – plan to discuss exploration of learning curve of treatment provider in one of our ongoing studies. |
| ID23 | Did not consider. |
| ID30 | Often time restrictions on completing. |
| ID32 | Unusually doesn’t change during the treatment period. |
| ID35 | I did a sensitivity analysis once to check for learning effects, but there was no evidence of it. I don’t know what other senior statisticians at our Unit have done. |
| ID39 | Not in the main analysis – aim for practitioner skill to have stabilised. |
