# Supplementary material for: Managing clustering effects and learning effects in the design and analysis of multicentre randomised trials: a survey to establish current practice
Source: Trials. 2020 May 27;21:433. doi: 10.1186/s13063-020-04318-x (PMC7251810; doi:10.1186/s13063-020-04318-x)
Supplement: Supplementary file 10 — Additional file 10: Supplementary Table 6. Comments on interaction investigation in example scenarios (Question 9). [file 13063_2020_4318_MOESM10_ESM.docx]

**Supplementary Table 6: Comments on interaction investigation in example scenarios (Question 9)**

| ID | Scenario | Stratification approaches | Free text |
| --- | --- | --- | --- |
| ID2 | A | No experience | Mainly worked on non-randomised trials in my Unit and so none of the above are applicable. |
|  | B | No experience |  |
|  | C | No experience |  |
| ID4 | A | Centre only  Neither centre nor treatment provider | For A, likely to investigate interaction for any stratification factors – so may include centre but, may be geographical region instead. |
|  | B | No experience |  |
|  | C | Centre only  Neither centre nor treatment provider |  |
| ID5 | A | Neither centre nor treatment provider | For A, not formally. For C, not formally. |
|  | B | Neither centre nor treatment provider |  |
|  | C | Neither centre nor treatment provider |  |
| ID6 | A | No response | For A, sometimes, specific to each trial. Depends on the nature of the intervention and numbers within treatment provider. For C, depends on numbers and nature of intervention |
|  | B | Neither centre nor treatment provider |  |
|  | C | No response |  |
|  | D | No response | For D, depends on numbers and nature of intervention. For e, depends on numbers and nature of intervention. |
|  | E | No response |  |
| ID7 | A | Centre only  Treatment provider only | For A, would usually (given sufficient numbers) look at centre*treatment group i/a; on a limited number of occasions looked at provider*treatment group i/a |
|  | B | Neither centre nor treatment provider |  |
|  | C | No experience |  |
| ID8 | A | Both centre and treatment provider | Some investigation is often useful - maybe qualitative. Rare to detect significant, clear interaction and therefore often summarise descriptively. |
|  | B | Centre only |  |
|  | C | Centre only |  |
|  | D | Both centre and treatment provider | Only have experience of one trials in Scenario D. We did an informal assessment for individual surgeon. |
|  | E | No experience |  |
| ID9 | A | Centre only | Centres are now nested within the treatment provider and makes a multilevel model. |
|  | B | Centre only |  |
|  | C | No experience |  |
| ID10 | D | No experience | For D, only one such trial in my experience – data collection and analysis plans still in development. |
|  | E | No experience |  |
| ID13 | A | Neither centre nor treatment provider | Has never been of interest. |
|  | B | Neither centre nor treatment provider |  |
|  | C | No experience |  |
| ID14 | A | Centre only  Treatment provider only | For A, maybe exploratory as usually not powered. For B, maybe exploratory as usually not powered. General comments: Trial by trial decision as appropriate in relation to interpretation and associated power. |
|  | B | Neither centre nor treatment provider |  |
|  | C | Treatment provider only |  |
|  | D | Centre only  Treatment provider only | For D, if specialist. For E, if specialist. General comments: Considered and decided at trial by trial basis - depends on phase and question. |
|  | E | Centre only  Treatment provider only |  |
| ID15 | A | Centre only  Treatment provider only | Might group in 'small' trials and do a treatment*county interaction for example. Often do subgroup analysis and plot treatment effect within site in a forest plot. |
|  | B | Neither centre nor treatment provider |  |
|  | C | Neither centre nor treatment provider |  |
| ID22 | A | Neither centre nor treatment provider | For A, most trials we have adjusted for the centre and treatment provider but not performed any treatment-by-centre interaction. |
|  | B | Neither centre nor treatment provider |  |
|  | C | Neither centre nor treatment provider |  |
| ID30 | D | Treatment provider only | Only limited experience in our Unit of d. |
|  | E | No experience |  |
| ID32 | A | Centre only | We usually look at centre by treatment interaction to assess whether the treatment effect is similar across centres but do not normally present this as part of the model. |
|  | B | Centre only |  |
|  | C | Centre only |  |
|  | D | Centre only | We usually look at centre by treatment interaction to assess whether the treatment effect is similar across centres but do not normally present this as part of the model. |
|  | E | Centre only |  |
| ID35 | A | Neither centre nor treatment provider | Providing best guess here. |
|  | B | Neither centre nor treatment provider |  |
|  | C | No experience |  |
| ID38 | A | Neither centre nor treatment provider | For A, I’ve put neither because we don’t routinely do it. I do usually work up a forest plot to explore within centre treatment effects, no formal treatment-by-centre interactions. |
|  | B | Neither centre nor treatment provider |  |
|  | C | No experience |  |
|  | D | Neither centre nor treatment provider | For D, again I’ve put neither because we don’t routinely do it. I do usually work up a forest plot to explore within centre treatment effects, no formal treatment-by-centre interactions, but I can’t recall drilling down to surgeon level in forest plots. |
|  | E | Neither centre nor treatment provider |  |
| ID39 | A | Neither centre nor treatment provider | For A, not done routinely – if interest in a centre effect in a particular trial, an investigation of the treatment by centre interaction would be pre-specified. For B, not done routinely, and would point out the problems of looking at interaction effects when sample size is small if the chief investigator requested such an analysis. |
|  | B | Neither centre nor treatment provider |  |
|  | C | No experience |  |
|  | D | Neither centre nor treatment provider | For D, not done routinely – if interest in a centre effect in a particular trial, an investigation of the treatment by centre interaction would be pre-specified. For E, not done routinely – if interest in a centre effect in a particular trial, an investigation of the treatment by centre interaction would be pre-specified. |
|  | E | Neither centre nor treatment provider |  |
