# Supplementary material for: Managing clustering effects and learning effects in the design and analysis of multicentre randomised trials: a survey to establish current practice
Source: Trials. 2020 May 27;21:433. doi: 10.1186/s13063-020-04318-x (PMC7251810; doi:10.1186/s13063-020-04318-x)
Supplement: Supplementary file 11 — Additional file 11: Supplementary Table 7. Details of how Units explore heterogeneity by centre in the presence of a treatment effect (Question 10a). [file 13063_2020_4318_MOESM11_ESM.docx]

**Supplementary Table 7: Details of how Units explore heterogeneity by centre in the presence of a treatment effect (Question 10A)**

| ID | Graphical display | Analytical methods | Further details on exploring heterogeneity by centre |
| --- | --- | --- | --- |
| ID1 | Yes | Yes | Depends |
| ID2 | Yes | Yes | Interaction tests done where necessary. |
| ID3 | Yes | No | We generally work with small studies so far and significance testing of treatment by centre would not seem appropriate or reliable with such small samples. |
| ID4 | No | Yes | Depends |
| ID6 | Yes | Yes | Depends |
| ID7 | Yes | Yes | Depends on whether any difference between centres is likely to be relevant of not. We would usually present some basic graphs and summary statistics at the centre level; sometimes pre-specified centre*treatment will be assessed, but always with the caveat of likely to have low power. In larger trials, sometimes presented Chief Investigators for the treatment effect by centre. |
| ID8 | Yes | Yes | Happened once only and we'd already planned to do this. This was a stepwise trial - hoping someone will publish at some point. Wanted to do this for fidelity assessment - qualitative. |
| ID10 | Yes | Yes | Graphical methods usually take precedence; supported by analytical methods. |
| ID14 | Yes | Yes | Depends |
| ID15 | Yes | Yes | One trial with a positive treatment effect explored variation by centre graphically and by descriptive statistics. This was not done due to positive treatment effect and was pre-planned. Another explored through forest plots - this has post hoc and in a study where there was no overall treatment effect. |
| ID18 | Yes | Yes | Forest plot and test. |
| ID19 | Yes | Yes | Tabulations, multivariate models, etc. |
| ID21 | Yes | No | Forest plots typically. |
| ID22 | Yes | Yes | Most of the trials have explored the heterogeneity of treatment effects across centres by either graphical or analytical methods. |
| ID23 | Yes | Yes | Not significance tests. |
| ID27 | Yes | No | Depends. Must by prespecified and of interest a priori. |
| ID30 | Yes | Yes | Treatment by centre interaction testing but rarely go beyond this - requires careful consideration of the implication of explanation to the Chief Investigator. |
| ID32 | Yes | No | Don't use significance tests as no power for these. |
| ID34 | Yes | No | Explore by region. |
| ID35 | Yes | No | Don't do this routinely but we probably do it a fair amount. If we did it, we would probably try to estimate treatment effect within each centre but this wouldn't be possible for tiny centres. |
| ID39 | Yes | Yes | Not done routinely - if interest in a centre effect in a particular trial, and investigation of the treatment by centre interaction would be pre-specified. If not pre-specified, we would not allow an interaction to be prompted by a positive treatment effect. |
| ID42 | Yes | Yes | Depends |
