# Supplementary material for: Managing clustering effects and learning effects in the design and analysis of multicentre randomised trials: a survey to establish current practice
Source: Trials. 2020 May 27;21:433. doi: 10.1186/s13063-020-04318-x (PMC7251810; doi:10.1186/s13063-020-04318-x)
Supplement: Supplementary file 12 — Additional file 12: Supplementary Table 8. Details of how Units explore heterogeneity by treatment provider in the presence of a treatment effect (Question 10b). [file 13063_2020_4318_MOESM12_ESM.docx]

**Supplementary Table 8: Details of how Units explore heterogeneity by treatment provider in the presence of a treatment effect (Question 10B)**

| ID | Effect explored | Graphical display | Analytical methods | Further details on exploring heterogeneity by treatment provider |
| --- | --- | --- | --- | --- |
| ID1 | No response | NA | NA | No experience in trials of this type. |
| ID2 | No response | NA | NA | No experience in trials of this type. |
| ID3 | No | No | No | Generally we do not consider treatment provider effects. After today, I will take this back to our Unit and see if we should start taking it into account in our designs and analyses. |
| ID5 | No response | NA | NA | No experience in trials of this type. |
| ID6 | Yes | Yes | Yes | Depends. |
| ID7 | Yes | Yes | Yes | The provider effect doesn't tend to be of particular interest in our trials, although I believe that in complex intervention trials, the provider effect can be underplayed. |
| ID8 | No | No | No | Too awkward - often we do course/group interventions with course leads swapping in/out/leaving etc. |
| ID14 | Yes | Yes | Yes | Sometimes, this is trial dependent - if relevant and interpretable. |
| ID35 | Yes | No | No | Need to be careful not to suggest some clinicians are worse than others (similarly for centres). |
| ID39 | No | No | No | Not done routinely and I don't think we have pre-specified this. |
| ID42 | Yes | Yes | Yes | Depends. |
